# Associations between bone mineral density, body composition and amenorrhoea in females with eating disorders: a systematic review and meta-analysis

**DOI:** 10.1186/s40337-022-00694-8

**Published:** 2022-11-18

**Authors:** Mariana P Lopes, Lauren Robinson, Brendon Stubbs, Marle dos Santos Alvarenga, Ligia Araújo Martini, Iain C Campbell, Ulrike Schmidt

**Affiliations:** 1 Nutrition Department, School of Public Health University of São Paulo, Av. Dr. Arnaldo, 715 - Cerqueira César, São Paulo, São Paulo 01246-904 Brazil; 2 Section of Eating Disorders, Department of Psychological Medicine, Institute of Psychiatry, Psychology and Neuroscience, King’s College London, 6 De Crespigny Park, London, SE5 8AF UK; 3 Maudsley Hospital, South London and Maudsley NHS Foundation Trust, Denmark Hill, London, SE5 8AZ UK

**Keywords:** Eating disorder, Anorexia nervosa, Bulimia nervosa, Bone mineral density, Osteoporosis, Body composition, Fat mass, Lean body mass, Amenorrhoea

## Abstract

**Background:**

Lower bone mineral density (BMD) increases the risk of osteoporosis in individuals with eating disorders (EDs), particularly women with anorexia nervosa (AN), making them susceptible to pain and fractures throughout adulthood. In AN, low weight, hypothalamic amenorrhoea, and longer illness duration are established risk factors for low BMD, and in people with other EDs a history of AN seems to be an important risk factor for low BMD.

**Purpose:**

To conduct a systematic review and meta-analysis of BMD in individuals with EDs, including AN, bulimia nervosa (BN), binge-eating disorder (BED) and other specified feeding or eating disorders (OSFED) compared to healthy controls (HC).

**Methods:**

Following PRISMA guidelines, electronic databases were reviewed and supplemented with a literature search until 2/2022 of publications measuring BMD (dual-energy X-ray absorptiometry or dual photon absorptiometry) in females with any current ED diagnosis and a HC group. Primary outcomes were spine, hip, femur and total body BMD. Explanatory variables were fat mass, lean mass and ED clinical characteristics (age, illness duration, body mass index (BMI), amenorrhoea occurrence and duration, and oral contraceptives use).

**Results:**

Forty-three studies were identified (N = 4163 women, mean age 23.4 years, min: 14.0, max: 37.4). No study with individuals with BED met the inclusion criteria. BMD in individuals with AN (total body, spine, hip, and femur), with BN (total body and spine) and with OSFED (spine) was lower than in HC. Meta-regression analyses of women with any ED (AN, BN or OSFED) (N = 2058) showed low BMI, low fat mass, low lean mass and being amenorrhoeic significantly associated with lower total body and spine BMD. In AN, only low fat mass was significantly associated with low total body BMD.

**Conclusion:**

Predictors of low BMD were low BMI, low fat mass, low lean mass and amenorrhoea, but not age or illness duration. In people with EDs, body composition measurement and menstrual status, in addition to BMI, are likely to provide a more accurate assessment of individual risk to low BMD and osteoporosis.

## Plain English summary

Individuals with eating disorders (EDs) have an increased risk for developing osteoporosis and suffering fractures. To better understand this problem, we conducted a systematic review and meta-analysis comparing bone mineral density (BMD) of females with EDs with that of healthy people without an ED. We also tried to identify key factors linked with reduced bone mass in EDs. We included studies reporting BMD of individuals with anorexia nervosa (AN), bulimia nervosa (BN), binge-eating (BED) or other non-specified ED (OSFED), and of healthy controls. We found that people with AN had overall lower BMD than controls and also in the spine, hip, and femur. In people with BN, there was lower BMD overall and in the spine, but that must be only in those who previously had AN. In people with OSFED, BMD was lower in the spine. Having a low BMI, low fat mass, low lean mass and not having menstrual periods seem to negatively affect BMD. Therefore, this systematic review supports the idea that people with current or past AN, irrespective of their current ED diagnosis, should have their bone health assessed. For early identification of those most at risk, body composition measurements, current menstrual status, duration of amenorrhoea and presence or absence of a history of AN should be considered in clinical practice.

## Background

Eating disorders (EDs) are characterised by aberrant eating patterns, significant psychopathology, distress and/or impairment [1]. Anorexia nervosa (AN) is associated with low weight, fear of gaining weight and over influence of weight or shape on self-judgment. Two subtypes of AN are distinguished: a restricting type (AN-R) and a binge-eating/purging type (AN-BP). Bulimia nervosa (BN) is characterised by dieting, binge-eating and compensatory behaviours (i.e. self-induced vomiting and laxatives or diuretics misuse), while in binge-eating disorder (BED), distressing episodes of loss-of-control eating are not followed by compensatory behaviours [1]. Other specified feeding or eating disorder (OSFED), previously called eating disorder not otherwise specified (EDNOS), is a diagnostic ‘hold-all’ category for all other EDs which do not meet the full diagnostic criteria for AN, BN or BED [1].

Eating behaviours and symptoms affect individuals’ growth, development, metabolism, and body composition (fat mass and fat-free mass) [2]. Decreased leptin secretion by a diminished adipose tissue inhibits the hypothalamic–pituitary–gonadal axis (HPG). This leads to low levels of oestrogen and testosterone, increased bone resorption, and decreased bone formation [3]. Osteoporosis and osteopenia are silent conditions, characterised by decreased bone mineral density (BMD), increasing the risk of fractures [4]. Women with EDs have higher risk of developing osteopenia [5], osteoporosis, and bone fractures [6, 7]. In a study with 130 women with AN, 54% had osteopenia and 38% osteoporosis [8]. A large Swedish study by Axelsson et al. [9] with over 9000 patients with EDs of both male and female sex, found risk of fractures increased irrespective of age and sex. Longitudinal studies of patients with AN by Lucas et al. [10] and Frølich et al. [11] show that the fracture risk remains elevated several decades after initial diagnosis and even after remission compared to that in healthy controls (HC).

In AN, low nutrient intake, excessive physical activity and purging behaviours are associated with BMD loss [12, 13]. Known risk factors are low weight, hypothalamic amenorrhoea, and longer illness duration [6, 14, 15], with low weight being one important predictor of osteoporosis and risk of fracture [12]. A previous meta-analysis showed lower BMD in individuals with BN than HC but only for those with a history of AN [14]. There is some temporal fluidity between these diagnoses, e.g. in one study 50–64% of women with AN experienced bulimic symptoms, and one-third of them crossed over from AN to BN when followed for 7 years [16]. However, the relationship between BMD in BED and OSFED/EDNOS, is less clear than in AN due to the paucity of studies [1, 6, 14].

Osteopenia and osteoporosis in EDs are difficult to treat [17], with limited pharmacological treatments [18]. Thus, early identification of those most at risk and targeted interventions are imperative for reducing the problem [18]. Previous systematic reviews and meta-analyses have shown lower BMD in individuals with AN than HC [6, 14, 19]. They also showed that lower BMD in AN was associated with lower fat-mass, fat-free mass [19] and longer duration of amenorrhoea [6]. Since the publication of DSM-5, in which amenorrhoea is no longer a criterion for AN diagnosis [1], multiple studies assessing BMD in EDs have been published. Therefore, there is a need to re-assess the literature, in relation to the potential role of amenorrhoea (presence and duration) and changes in body composition in the lowering of BMD in females with EDs, not only AN.

The aim of this study was to conduct a systematic review and meta-analysis of BMD by dual-energy X-ray absorptiometry (DXA) or dual photon absorptiometry (DPA) across commonly measured anatomical sites (total body, spine, hip, and femur) in females with any EDs (AN, both AN-R and AN-BP subtypes, BN, BN with a history of AN, BED, and OSFED/EDNOS) in comparison to HC. Secondly, we wanted to identify predictors of the difference in BMD between EDs and HC by means of meta-regression analyses involving age, illness duration, body mass index (BMI), fat mass, lean mass, occurrence and duration of amenorrhoea, and oral contraceptive pill (OCP) use.

## Materials and methods

### Study selection

This is a systematic review of the international literature based on searches of MedLine, EMBASE, and PsychoInfo following the PRISMA guidelines [20] and registered in PROSPERO (CRD42019122053). Amendments were made in the registered protocol in order to conduct a meta-analysis of BMD, assessed by DXA scan in individuals with EDs (inclusion of BED and OSFED/EDNOS diagnoses and adolescents) and to identify predictors of BMD (secondary outcomes were limited to fat mass, lean mass, illness duration, BMI, amenorrhoea occurrence, duration of amenorrhoea and oral contraceptive use). Manual searches were conducted, and reference lists were searched for relevant articles. The following indexed descriptors were used and their combination for EDs (Anorexia nervosa*, Bulimia nervosa*, Eating disorders*, Binge-eating disorder*, EDNOS*, OSFED*) and bone health (Bone density*, Bone mineral density*, Bone mineral content*, Bone mass*, Fracture* and Osteoporosis*).

### Inclusion and exclusion criteria

Publications in English were included if they were studies of adolescent (> 12 years) and adult females with a current ED diagnosis (AN, BN, BED or OSFED/EDNOS) and a HC group, and measured BMD using DXA or DPA. The BN and OSFED/EDNOS groups included individuals with or without a history of AN. Studies with participants recruited from the same population, institution and/or period had an independent sample selected only. Selection criteria were, in order: larger sample size, most recent publication date, and most recent ED diagnostic criteria adhered to upon recruitment. Study design (i.e. longitudinal, cross-sectional) was not used as an inclusion criterion, but only baseline measures of BMD were included.

Studies using other methods for bone and body composition assessment, not including BMD, such as skinfold measurement, bioelectrical impedance analysis (BIA) and computerised tomography (CT) scan, magnetic resonance imaging (MRI) were not included. The final search was performed on 2/2022. Following exclusion of duplicates, articles were screened based on title and abstract and were excluded if they were reviews or did not match inclusion criteria. Original manuscripts were assessed by two independent reviewers (MPL and LR): disagreements were resolved by consensus or with a third co-author.

### Data extraction

Information extracted from each included paper consisted of: (1) article reference (study name, authors, year of publication, and country); (2) characteristics of participants with EDs and control group (number of participants, age, mean BMI, ED diagnosis, method of diagnosis, number of participants with amenorrhoea, duration of amenorrhoea, numbers using OCP, illness duration; sample source); (3) outcome measures of interest: DXA or DPA scanning methods, BMD data (total body, spine, hip, and femur), fat mass and lean mass where available.

### Quality assessment

The Newcastle–Ottawa scale (NOS) [21] was used for risk of bias assessment. This allows quality evaluation of non-randomised studies for meta-analyses based on: selection of the study group, group comparability, and ascertainment of exposure of interest for case–control studies. It includes the definition of cases and controls, method of control selection, representativeness of cases, and comparability of cases and controls. Exposure of interest is assessed as the diagnosis of the ED and the sample bias through non-response rate. For case–control studies, a NOS score below 5 suggests a high risk of bias (maximum of 9) [22].

### Statistical analysis

Statistical analyses were conducted on STATA SE software, version 16 [23]. Meta-analyses were performed using ‘meta set’ and ‘meta-summarize’ commands with random-effect models of total body, spine, femur, and hip BMD (where available) of ED groups and HC due to suspected heterogeneity. Standardised mean difference (SMD) was the primary outcome of the meta-analyses, generated by the ‘meta summarize’ command. Forest plots were generated using the ‘meta forest’ and funnel plots using the ‘meta funnel’ commands.

Meta-regression analyses were performed using the ‘metareg’ command to investigate the association between age, BMI, fat mass, lean mass, percentage of participants with amenorrhoea, percentage using OCP, and illness duration and the SMD of total body, spine, femur, and hip BMD (in the whole ED group and in the AN group).

Due to the limited reporting of AN subtypes, different methods of determining ED diagnoses, and cross-over between ED diagnoses, heterogeneity was suspected. Heterogeneity was assessed using the Higgins I^2^ test, with > 25% as low, > 50% as moderate, and > 75% as high. Trim and fill correction was used to investigate potential publication bias [24]. An Egger test was applied to investigate small-study effects on the spine, femur, hip, and total body BMD [25].

## Results

### Search results

**Fig. 1:**
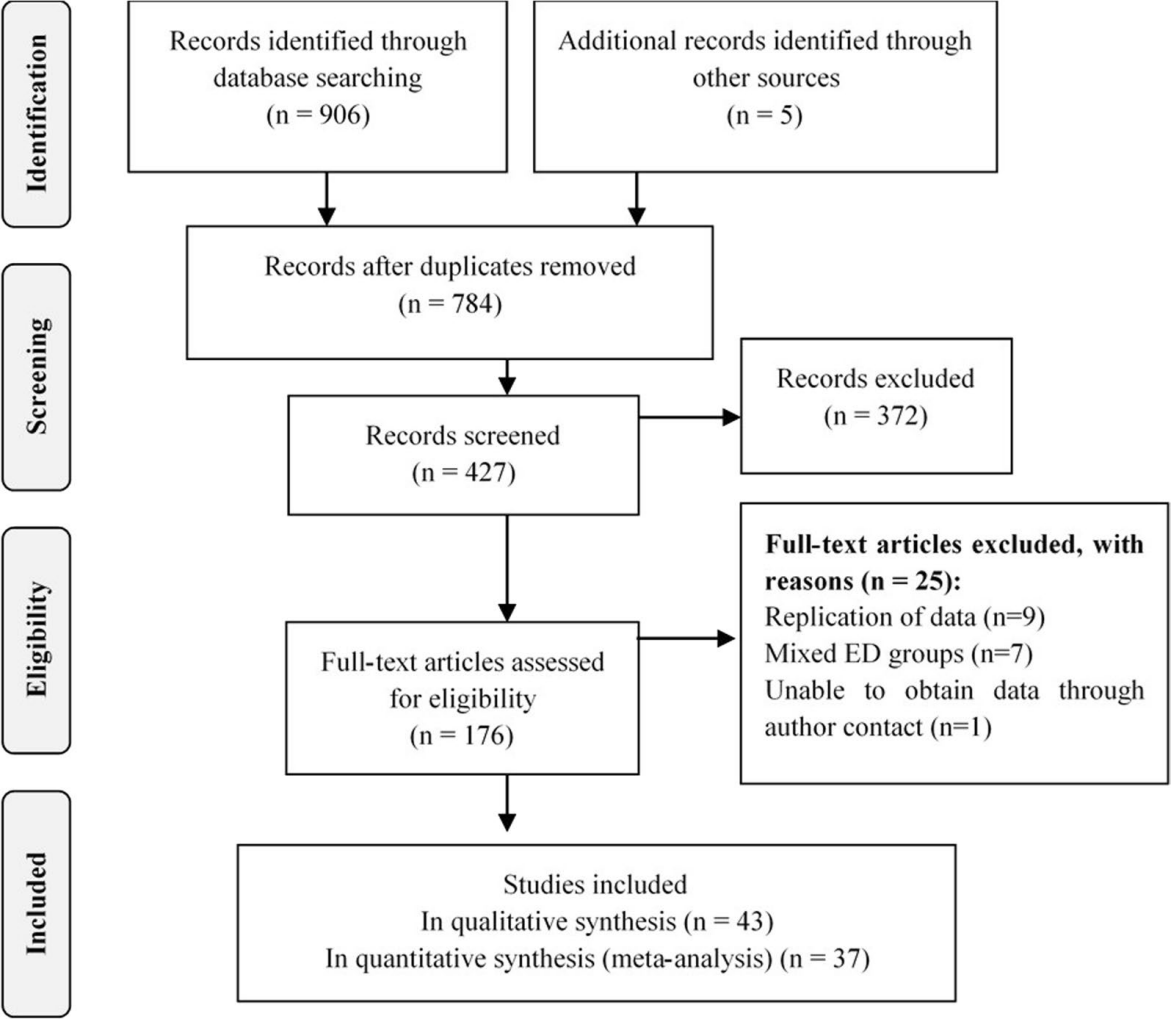
Flow diagram of studies included in the systematic review and meta-analysis

In the electronic database search, 911 full-text articles were found, resulting in 784 original articles after removal of duplicates. Following screening of titles and abstracts, 372 studies were excluded according to the following criteria: review or book, participants being male, no control group, females with athlete’s triad syndrome and exercisers, other diseases or non-DXA or DPA assessment or being a case-report. 176 full-text articles were reviewed and 43 original articles were included in the qualitative review and 37 in at least one of four meta-analyses (Fig. 1).

### Study characteristics

Publication dates ranged from 1989 to 2022. Literature sources were: USA (42.9%); Europe (40.5.5%), Australia and New Zeland (7.1%) and Asia (9.5%).

In total, 43 papers were included with 4163 participants from 61 different groups with AN, BN or OSFED/EDNOS, and 43 healthy control groups. Participants mainly had AN diagnoses (N = 45 studies, 73.8% of the ED sample), with the remainder having BN (N = 15 studies, 24.6% of the ED sample) or OSFED/EDNOS diagnoses (N = 2 studies, 1.6% of the ED sample). No study with individuals with BED compared either to HC or to individuals with BED and a previous history of AN met the inclusion criteria. Of the 61 EDs groups included in this review, 50.8% (31/61) were described as ‘AN’ and 13.1% (8/61) as ‘BN’. In relation to AN subtypes, 12.7% (8/61) of the groups were of the ‘AN-Restrictive subtype (AN-R)’ and 1.6% (1/61) the ‘AN-Binge Purge subtype (AN-BP)’. In 6.5% (4/61) of them, ‘BN with no previous AN history (BN-NPAN)’ and in 3.3% (2/61) ‘BN with previous AN (BN-PAN)’ were reported. The diagnosis of ‘AN with an additional diagnosis of BN (according to DSM-III-R) (AN-BN)’’ was reported in 3.3% (2/61) of the groups. ‘OSFED/EDNOS’ and ‘AN-Atypical’ corresponded to 1.6% (1/61) each of the groups included.

From the 61 EDs groups, data on BMD were available for the total body (N = 35 groups), spine (N = 54 groups), hip (N = 19 groups) and femur (N = 24 groups) in AN, BN and OSFED groups. Due to the limited BMD measures in BN and OSFED groups for the hip (N = 2), whole ED group (AN, BN and OSFED) analyses were conducted for the total body, spinal and femur regions, and for the hip (N = 19) with AN groups only.

#### AN

Participants with AN had a mean age of 22.4 years [14.6–34.7], a BMI of 16.3 kg/m^2^ [14.4–19.1], an illness duration of 4.7 years [0.7–13.0] and 17.4 months of amenorrhoea [1.5–46.5]: 62.21% were amenorrhoeic and 24.4% used OCP. The majority of the studies (48.9%) used DSM-IV for diagnosis, followed by DSM-III-R (26.7%), DSM-5 (11.1%), ICD-10 (6.7%), both DSM-IV/5 (4.4%) and one study did not report it. The number of groups in the studies with investigation of the different parameters were: total body BMD (n = 26, 57.8%), spine (n = 38, 84.4%), femur (n = 21, 46.7%), hip (n = 19, 42.2%), fat mass (n = 28, 62.2%) and lean mass (n = 27, 60.0%).

#### BN

The BN group had a mean age of 25.6 years [20.7–30.7], a BMI of 21.5 kg/m^2^ [19.0–22.3], an illness duration of 8.1 years [2.8–16.6], and a duration of amenorrhoea (reported in three studies only and in one of them with participants with a previous diagnosis of AN) of 62.4 months [19.1–132.0]: 35.7% were amenorrhoeic and 35.7% were using OCP for contraception or for any other reason. Most studies (57.1%) used DSM-IV for diagnosis, followed by DSM-III-R (28.6%) and DSM-5 (14.3%). Body composition measurements investigated in the BN groups were: total body BMD (n = 7, 50.0%), spine (n = 14, 100.0%) femur (n = 3, 21.4%), hip (n = 2, 14.3%), fat mass (n = 7, 50.0%) and lean mass (n = 2, 14.3%).

#### OSFED/EDNOS

Only two studies included individuals with OSFED (EDNOS or atypical AN), with a mean age of 21.0 years [14.5–27.6] and a BMI of 16.9 kg/m^2^ [15.6–18.3] [26, 27]. In the study by Bratland-Sanda et al. [27], 36% of the individuals reported a history of AN. Duration of illness and amenorrhea were reported by one of these studies (Table 1). One paper used DSM-IV [27] for diagnosis and the other [26] the DSM-5 criteria. Body composition measurements assessed were: total body BMD (n = 2), spine (n = 2), femur (n = 1), hip (n = 0), fat mass (n = 1), and lean mass (n = 1). The study by Bacopoulou et al. [26] did not present standard deviations from BMD measures, therefore was not included in the whole ED meta-analysis and meta-regressions.

**Table 1:** Summary of study characteristics (n = 43)

Author	Country	ED	Sample size (N)	BMI (SD) (kg/m^2^) or % of IBW	Amenorrhoea (%)	OCP use (%)	Diagnostic method	Design	ED population	Controls	Scanning method	Bone density outcome measures	Body composition outcome measures	NOS-Scale stars of 9
Andersen et al. [28]	USA	AN-R	28	68.1 (14.2) % of IBW	Yes	Unknown	DSM-IV	Case–control	Hospital outpatients	Similar age and white controls Referred to radiology for non-orthopaedic, non-eating disorder problems to ‘rule out’ osteoporosis	DXA	Spine BMD		5
					Unknown %									
		AN-BP	36	73.3 (11.7) % of IBW	Unknown	Unknown								
		BN-PAN	18	111.3 (27.8) % of IBW	Unknown	Unknown								
		BN-NPAN	14	109.4 (11.3) % of IBW	Unknown	Unknown								
		HC	15	Unknown	Unknown	Unknown								
Bachmann et al. [29]	USA	AN	65	15.9	Yes	Yes	DSM-5	Case–control	Premenopausal women who had undergone quantitative computed tomography	Premenopausal women who had undergone quantitative computed tomography	DXA QCT	Spine BMD	Fat mass Lean mass	5
					Unknown %	Unknown %								
		HC	45	19.8										
Bacopoulou et al. [26]	GRE	AN	17	17.0 (1.1)	Yes	Unknown	DSM-5	Case–control	Adolescent medicine clinic	Healthy controls Age-matched girls presented for annual health examination in an adolescent medicine clinic	DXA	Total body BMD Spine aBMD		5
					100%									
		AN-Atypical	5	18.3 (16.0–22.9)	Yes	Unknown	DSM-5							
					60%									
		HC	15	22.1 (1.7)										
Bratland-Sanda et al. [27]	NOR	AN	8	15.6 (1.4)	Unknown	Unknown	DSM- IV	Case–control	ED hospitalised patients	Healthy controls Age-matched Randomly selected from a national pool	DXA	Total body BMD Spine BMD Femoral neck BMD	Fat mass Lean mass	8
		BN	29	22.3 (3.5)	Unknown	Unknown	DSM- IV							
		EDNOS	22	21.0 (3.2)	Unknown	Unknown	DSM- IV							
		HC	53	25.3 (4.8)										
Bredella et al. [30]	USA	AN	10	18.4 (1.0)	Yes	Yes	DSM-IV	Case–control	ED clinics	Healthy controls Recruited through clinic advertisements	DXA	Total body BMD Spine BMD Femoral BMD Hip BMD	Fat mass Lean mass	7
					100%	20%								
		HC	10	20.5 (2.6)										
Bredella et al. [31]	USA	AN	10	17.6 (1.0)	Unknown	Unknown	‘Psychiatric diagnostic criteria for AN’	Case–control	Clinic referrals	Healthy controls Recruited through community advertisements	DXA MRI	Total body BMD Spine BMD Hip BMD		5
		HC	10	21.9 (1.7)										
Bredella et al. [32]	USA	AN	5	18.3 (0.9)	Unknown	No	DSM-IV	Case–control	Clinic referrals	Healthy Controls Recruited through community advertisements	DXA Fluorodeoxyglucose- PET CT	Total body BMD Spine BMD Femoral neck BMD Hip BMD Lateral spine BMD	Fat mass Lean mass	5
		HC	5	21.9 (1.7)										
Čagalová et al. [33]	SLO	AN	63	14.5 (1.8)	Yes	No	DSM-5	Case–control	Hospital patients of the Department of Paediatrics	General paediatric practice	DXA	Total body BMD Spine BMD Hip BMD		5
					81.4%									
		HC	20	20.2 (1.7)										
Davies et al. [34]	USA	AN	26	Unknown	Yes	Yes	DSM-III-R	Case–control	Clinic records of eating disorder patients from medical centre	Healthy controls Select for fitness and diet study or for a DXA and DPA compar ison group	DPA	Spine BMD Forearm BMD		4
					80.8%	15.4%								
		AN-BN	26	Unknown	Yes	Yes	DSM-III							
					61.5%	23.1%								
		BN	11	Unknown	Yes	Yes								
					18.2%	9.1%								
		HC	211	Unknown										
Estour et al. [35]	FRA	AN-R	40	16.0 (0.8)	Yes	No	DSM-IV	Case–control	Hospital outpatients	Healthy controls ranging 18.6–25.0 kg/m2 BMI	DXA	Spine BMD Femoral BMD	Fat mass Lean mass	5
					100%									
		HC	54	20.9 (2.2)										
Faje et al. [36]	USA	AN	21	17.8 (0.2)	Unknown	Unknown	DSM-IV	Cohort	Hospital outpatients	Healthy controls with normal weight	DXA	Total body aBMD Spine aBMD Hip aBMD Distal radius aBMD	Fat mass Lean mass	6
		HC	23	22.4 (0.5)										
Fazeli, Klibanski [37]	USA	AN	26	16.7 (0.5)	Yes	No	DSM-IV	Cohort	ED referrals	Healthy controls Recruited through community advertisements	DXA MRI	Spine BMD Femoral BMD Hip BMD		5
		HC	20	22.6 (0.3)										
Fernández-Soto et al. [38]	SPA	AN-R	16	16.8 (1.5)	Yes	No	DSM-IV	Cohort	Clinic outpatients	Healthy controls Caucasian women	DXA MRI	Total body BMD Spine BMD	Fat mass Lean mass	5
		AN-R	31	19.1 (2.0)	Yes	No	DSM-IV							
		HC	25	21.8 (0.9)										
Frølich et al. [39]	DEN	AN	25	16.2 (1.3)	Yes	Yes	ICD-10	Case–control	ED unit outpatients	Healthy controls Age and height-matched females Randomly selected from a national pool	DXA	Spine BMD Femoral BMD Hip BMD	Fat mass Lean mass	7
					100%									
		HC	25	22.8 (2.7)										
Guo et al. [40]	CHI	AN	26	16.3 (0.6)	Yes		DSM-IV	Cohort	Clinical psychiatrist referrals	Healthy controls Age-matched	DXA	Total body BMD Spine BMD Hip BMD	Fat mass Lean mass	5
					100%									
		HC	24	20.8 (0.7)										
Haas et al. [41]	GER	AN	103	16.5 (1.6)	Yes	Yes	DSM-IV	Longitudinal observational	Patients of adolescent and youth medicine clinic	Normal weight controls Recruited from a local high school	DXA	Total body BMD	Fat mass Lean mass	5
					94.2%	5.6%								
		HC	51	20.8 (1.8)										
Iketani et al. [42]	JAP	AN	22	14.4 (2.3)	Yes	No	DSM-III-R	Cohort	Inpatients and outpatients	Healthy controls Age-matched healthy females	DPA	Total body BMD Spine BMD		5
					Unknown %									
		AN-BN	23	14.4 (1.6)	Yes	No	DSM-III-R							
					Unknown %									
		BN-PAN	10	19.0 (2.6)	Yes	No	DSM-III-R							
					40%									
		HC	10	19.5 (0.8)										
Kandemir et al. [43]	USA	AN	262	17.2 (0.1)	Yes		DSM-IV / DSM-5	Case–control	AN participants from Childhood Study	Healthy controls participants from Childhood Study	DXA	Total body BMD Spine BMD Hip BMD		5
					85.9%									
		HC	90	21.0 (0.2)										
Karlsson et al. [44]	AUS	AN	77	15.6 (0.2)	Unknown	Unknown	ICD-10	Case–control	AN patients untreated with oestrogen therapy	Healthy controls	DXA	Spine BMD Femoral neck BMD	Fat mass Lean mass	5
		HC	205	23.1 (0.3)										
Kooh et al. [45]	JAP	AN	22	15.9 (2.2)	Yes	Yes	DSM-III-R	Cohort	Clinic referrals: Adolescent medicine clinic	Healthy controls School and university students, no oral contraceptives	DXA	Spine BMD Femoral neck BMD	Fat mass Lean mass	5
					72.3%	4.50%								
		HC	24	21.6 (2.2)										
Maïmoun et al. [46]	FRA	AN-R	206	15.8 (1.7)	Yes	No	DSM-IV	Case–control	Hospital outpatients	Healthy controls Recruited through community advertisement	DXA	Total body aBMD Spine aBMD Hip BMD Proximal femur aBMD	Fat mass Lean mass	6
					100%									
		AN-R	99	16.3 (1.4)	No	Yes	DSM-IV							
						100%								
		HC	121	21.6 (2.3)										
Masala et al. [47]	ITA	AN	17	17.5 (1.6)	Unknown	Unknown	ICD-10	Cohort	Patients in weight gain programme	Healthy controls Exclusion included medication or illness to affect bone	DXA QCT	Spine BMD		4
		HC	27	24.3 (4.8)										
Mathisen et al. [48]	SWE	BN-NPAN	100		Unknown	Unknown	DSM-5	Cross-sectional	Patients from a randomised controlled trial	No healthy control group	DXA	Total body BMD Spine BMD	Fat mass Lean mass	6
		BN-PAN	37		Unknown	Unknown	DSM-5							
Misra et al. [49]	USA	AN	23	16.7 (1.2)	Unknown	Unknown	DSM-IV	Cohort	Clinic referrals	Healthy controls Age-matched and bone age- matched Recruited through adverts through healthcare providers and newspapers	DXA	Spine BMD Hip BMD	Fat mass Lean mass	6
		HC	21	21.7 (3.7)										
Misra et al. [50]	USA	AN	17	16.7 (1.3)	Unknown	Unknown	DSM-IV	Cohort	Paediatrician referrals	Healthy controls Age-matched and bone age- matched Recruited through mailings to paediatricians	DXA	Total body BMD Spine BMD Femoral neck BMD Hip BMD	Fat mass Lean mass	6
		HC	19	21.8 (3.4)										
Misra et al. [51]	EUA	AN	110	17.4 (0.1)	Yes	No	DSM-IV	RCT	Hospital outpatient treatment programme	Healthy controls Recruited through mailings to paediatricians	DXA	Spine BMD Hip BMD	Fat mass Lean mass	7
					100%									
		HC	40	21.4 (0.5)										
Morris et al. [52] ﻿	UK	AN	51	15.1 (1.9)	Yes	Yes	DSM-IV	Cohort	ED specialist referrals	Control group data from department of medical physics	DXA X-Ray	Total body BMD Spine BMD	Fat mass	5
					100%									
		BN	47	21.9 ( 3.4)		Yes	DSM-IV							
		BN-NPAN	26	22.3 (2.7)		Yes	DSM-IV							
		BN-PAN	21	21.4 (3.0)		Yes	DSM-IV							
		HC	40	23.3 (4.4)										
Naessén et al. [53]	SWE	BN	77	22	Yes	No	DSM-IV	Cohort	Recruited from hospital advertisements	Healthy controls Hospital advertising: hospital staff and students, no current diseases or medication prior to 3 months before the study	DXA	Total body BMD Spine BMD Leg BMD	Fat mass Lean mass	8
					7.8%									
		BN-NPAN	59				DSM-IV							
		BN-PAN	18				DSM-IV							
		HC	56	22.2										
Newman, Halmi [54]	USA	AN	18	73.2 (8.4)	Yes	No	DSM-III-R	Cross-sectional	Inpatients from an ED unit	Normal weight women	DXA	Spine BMD Femoral BMD		4
					100%									
		BN	12		Yes	Yes	DSM-III-R							
					66.7%	8.4%								
		HC	12											
Newton et al. [55]	AUS/UK	BN	20	21.8 (3.5)	Yes	Yes	DSM-III-R	Cohort	ED outpatient treatment programme	Healthy controls Age and sex-matched controls from hospital staff notice boards	DXA	Spine BMD		7
					20%									
		HC	16	21.9 (1.8)										
Olmos et al. [56]	SPA	AN	51	17.3 (2.4)	Yes		DSM-IV	Prospective longitudinal cohort study	ED unit outpatients	Healthy controls Hospital advertisements	DXA	Spine BMD Femoral neck BMD Hip BMD		5
					100%									
		HC	40	21.8 (2.7)										
Poet et al. [57]	FRA	AN	18	Unknown	Yes	No	DSM-III-R	Cohort	Hospital outpatients	Healthy controls Volunteers	DXA	Spine BMD		5
					100%									
		HC	36	Unknown										
Resch et al. [58]	AU	AN	20	16.0 (13–18)	Unknown	Unknown	DSM-III-R	Cohort	Hospital outpatients	Healthy controls Age-matched nursing school students	DXA	Spine BMD Hip BMD		5
		HC	20	23.0 (19–29)										
Schorr et al. [59]	USA	AN	46	16.7 (1.8)	Yes	Yes	DSM-5	Cross-sectional	Patients of National Institutes Health trials	Lean healthy controls From the National Institutes Health trial	DXA HRpQCT	Spine BMD Femoral BMD Hip BMD		6
					60.9%	15.3%								
		HC	29	22.6 (1.4)										
Seeman et al. [60]	AUS	AN	12	15.9 (1.3)	Yes	No	DSM-III-R	Cohort	Patients with AN	Healthy controls Volunteers with no illness that affects the bone, no drugs, no medication	DXA	Total body BMD Spine BMD Proximal femur Femoral neck BMD Ward’s triangle BMD Trochanter BMD	Fat mass Lean mass	4
					100%									
		AN	37	16.6 (0.4)	Yes	No	DSM-III-R							
					100%									
		AN OCP	16	17.2 (0.8)		Yes	DSM-III-R							
						100%								
		HC	52	23.3 (0.5)										
Singhal, Bredella [61]	USA	AN	55	18.7 (0.2)	Yes	No	DSM-IV / DSM-5	Cross-sectional	Ongoing studies assessing bone outcomes	Normal-weight controls From ongoing studies assesing bone outcomes	DXA HRpQCT	Total body BMD Spine BMD Femoral BMD Hip BMD	Fat mass Lean mass	6
					27.3%									
		HC	48	21.7 (0.3)										
Soyka et al. [62]	USA	AN	19	16.5 (0.4)	Yes	No	DSM-IV	Cohort	Healthcare provider referrals	Healthy controls Recruited through advertisement in primary care providers and newspapers, BMI 25th-90th percentile, one participant in pre- menarche	DXA	Total body BMD Spine BMD Lateral spine BMD	Fat mass Lean mass	6
					73.7%									
		HC	19	21.8 (0.4)										
Strumila et al. [63]	FRA	BN-NPAN	50	21.9 (2.6)	Irregular menstrual cycle	Unknown	DSM-5	Cross-sectional	ED unit	No	DXA	Spine BMD Hip BMD	Fat mass	5
					35%									
		BN-PAN	35	21.9 (2.9)	Irregular menstrual cycles	Unknown	DSM-5							
					46.7%									
Sundgot-Borgen et al. [64]	NOR	AN	13	15.8 (0.9)	Yes	No	DSM-IV	Case–control	Clinic referrals	Healthy controls University information board recruitment Comprehensive inclusion criteria for dietary, exercise and ED symptoms	DXA	Total body BMD Spine BMD Femoral neck BMD Leg BMD Arm BMD	Fat mass	7
					100%									
		BN	43	20.7 (2.0)	Yes	No	DSM-IV							
					32.6%									
		HC	17	22.0 (2.4)										
van Marken Lichtenbelt et al. [65]	NL	AN-R	12	16.5 (1.7)	Yes	Yes	DSM-III-R	Cohort	Non-hospitalised outpatients	Healthy controls Normal weight participating from a study on energy expenditure	DXA	Total body BMD	Fat mass Lean mass	3
					58.3%	33.3%								
		HC	16	23.7 (2.1)										
Walsh et al. [66]	USA	AN	8	17.4 (1.4)	Unknown	Unknown	DSM-IV	Cohort	Hospital outpatients	Healthy controls 90–100% ideal weight for age	DXA CT	Spine BMD Femoral neck BMD Hip BMD	Fat mass Lean mass	4
		HC	6	24.4 (2.5)										
Wojcik et al. [67]	USA	AN	15	17.6 (0.2)	Unknown	No	DSM-IV	Cohort	Healthcare referrals and community adverts	Healthy controls Recruitment through community advertisement	DXA	Total body BMD Spine BMD Femoral neck BMD Hip BMD	Fat mass Lean mass	4
		HC	16	23.5 (0.6)										
Wu et al. [68]	CHI	AN	25	17.5 (1.3)	Yes	Unknown	DSM-5	Case–control	ED providers referrals	Healthy controls Recruited through mailings to paediatricians	DXA	Total body BMD Spine BMD Femoral neck BMD	Fat mass Lean mass	4
					100%									
		HC	31	20.2 (1.3)										

*ED* eating disorder, *HC* healthy controls, *BMI* body mass index, *IBW* ideal body weight, *SD* standard deviation, *AN* anorexia nervosa, *BN* bulimia nervosa, *AN-R* AN-restrictive subtype, *BN-NPAN* BN with no previous AN history, *BN-PAN* BN with previous AN, *AN-BN* AN with an additional diagnosis of BN (according to DSM-III-R), *AN-PB* AN-binge purging subtype, *EDNOS* ED not otherwise specified, *OCP* oral contraceptive pill, *DSM* Diagnostic and Statistical Manual of Mental Disorders, *CID* International Classification of Diseases, *DXA* dual-energy X-ray absorptiometry, *DPA* dual photon absorptiometry, *CT* computerised tomography, *MRI* magnetic resonance imaging, *PET* positron emission tomography, *BMD* bone mineral density

#### HC

The HC group had a mean age of 23.7 years [14.0–37.4] and a BMI of 21.9 kg/m2 [19.5–24.4]. They were females, with no history of ED, from the same community as the cases (n = 30) [27, 30–37, 39–41, 44–47, 49–53, 55–59, 61, 62, 64, 65, 67] or hospital controls (n = 6) [26, 28, 43, 48, 63, 68]. Six studies did not provide information on selection of controls [29, 38, 42, 54, 60, 66]. HC were matched for age (n = 15) [26–29, 31, 33, 37, 41, 42, 46, 47, 55, 56, 58, 64, 68], age and BMI with BN participants [48, 53], age and bone age (n = 1) [49], age and fat mass (n = 1) [40], age and ethnicity (n = 1) [45] and age, lean mass and body fat (n = 1) [44].

Of the included studies, 14 adjusted their analysis for at least one of the following: age, race, BMI, bone age, height, weight, normal weight, age at menarche, fat and fat-free mass or sexual maturity [30, 36, 38, 39, 43, 50–52, 59–62, 66, 67]. Seven studies did not mention any adjustments for the control group [32, 34, 35, 54, 57, 63, 65].

### Quality assessment

Quality assessment of the studies is presented in Table 1. NOS scale scores ranged from 3 to 8 out of 9, with a mean of 5.3. Across all 43 studies, eight had a high risk of bias (score < 5) [34, 47, 54, 60, 65–68]. All of these had a cohort design or a publication date before the 1990’s. However, selection of participants was valid and adequate. Exposure assessment revealed only a few studies with the same methodology for ascertainment of ED and non-ED diagnosis, and none of the studies reported a non-response rate. The NOS score was not used to exclude articles from this review.

### Meta-analysis results

#### BMD in EDs

**Fig. 2:**
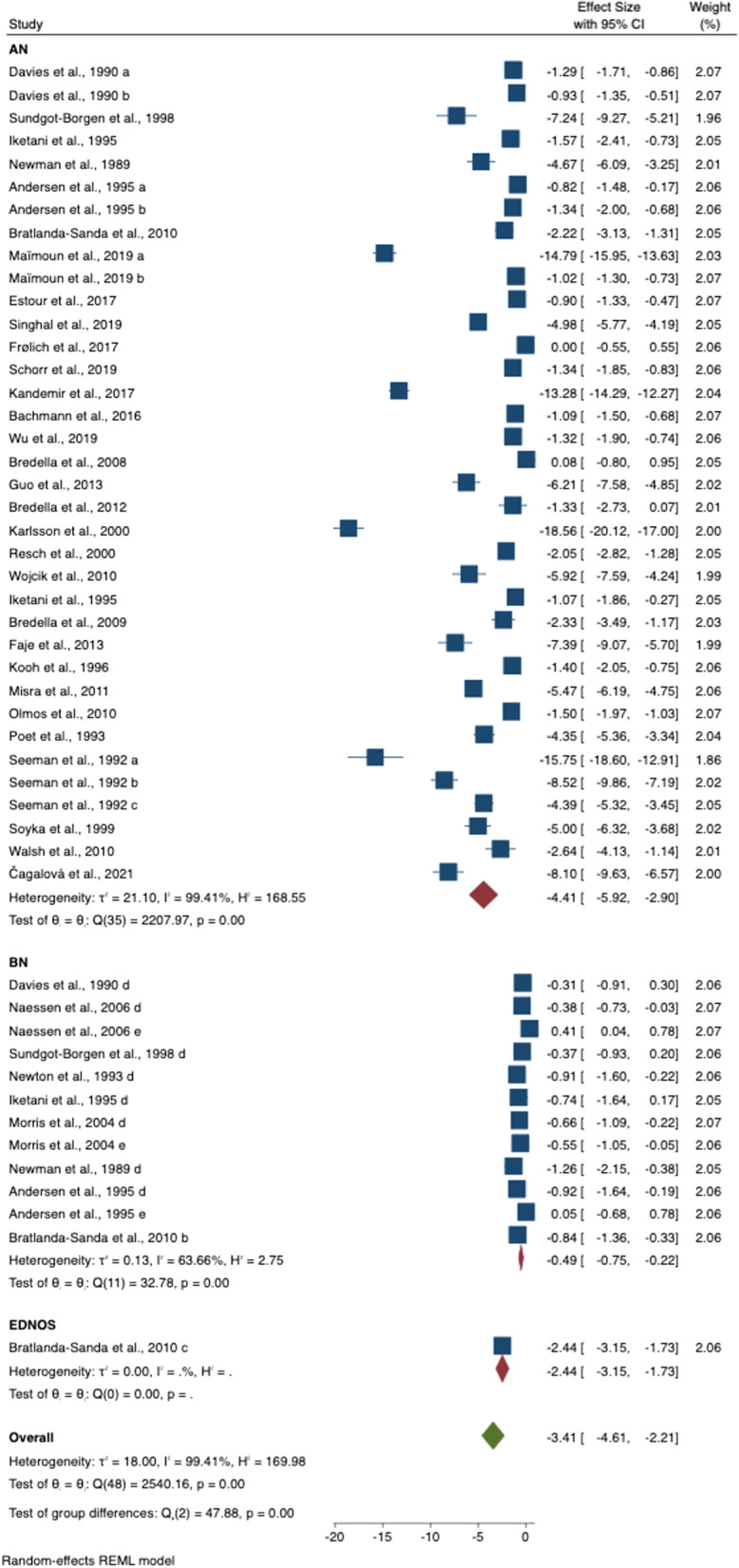
Meta-analysis results of spine bone mineral density in eating disorder (EDs) versus healthy controls (HC)

**Fig. 3:**
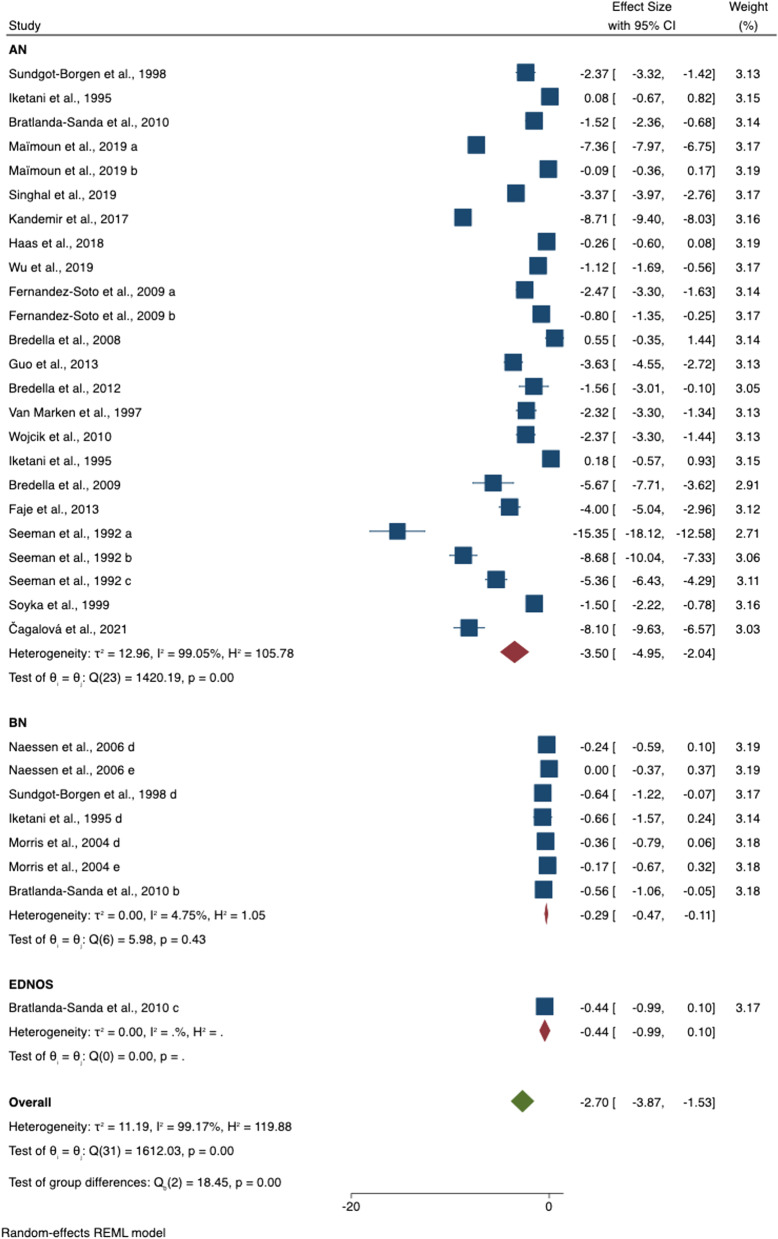
Meta-analysis results of total body bone mineral density in eating disorder (EDs) versus healthy controls (HC)

In the whole group analyses of AN, BN and OSFED/EDNOS (compared to HC), BMD was lower at three sites, total body BMD (SMD = − 2.62 [− 3.39 to − 1.84], *p* < 0.001), spine BMD (SMD = − 3.31 [− 3.98 to − 2.63], *p* < 0.001) and femur (SMD = − 3.08 [− 4.33 to − 1.83], *p* < 0.001). Hip BMD was only measured in studies of AN groups and is reported in the next section (Table 2 and Figs. 2, 3).

**Table 2:** Meta-analysis results of females with eating disorders (EDs) versus healthy control (HC) groups

Anatomical site	Groups in studies (N)	ED (N)	HC (N)	SMD	L 95% CI	U 95% CI	Z	*p*	*I*^2^ (%)	*Egger*	*p*
*All studies*											
Total body BMD	32	996	963	− 2.62	− 3.39	− 1.84	6.59	** < 0.001**	99.2	− 5.76	< 0.001
Spine BMD	49	1941	2299	− 3.31	− 3.98	− 2.63	9.61	** < 0.001**	99.4	− 6.87	< 0.001
Femur BMD	24	894	1051	− 3.08	− 4.33	− 1.83	4.82	** < 0.001**	98.7	− 5.03	< 0.001
*AN*											
Total body BMD	24	683	676	− 3.45	− 4.57	− 2.33	6.04	** < 0.001**	99.1		
Spine BMD	36	1553	1743	− 4.34	− 5.27	− 3.40	9.11	** < 0.001**	99.4		
Femur BMD	20	788	954	− 3.38	− 5.30	− 2.22	4.78	** < 0.001**	98.8		
Hip BMD	19	1078	688	− 4.95	− 6.78	− 3.12	5.30	** < 0.001**	98.9	− 2.52	0.012
*BN*											
Total body BMD	7	291	253	− 0.29	− 0.46	− 0.12	3.27	**0.001**	4.8		
Spine BMD	12	366	522	− 0.49	− 0.77	− 0.21	3.45	**0.001**	63.7		
Femur BMD	3	84	63	− 0.19	− 0.80	− 0.43	0.60	0.552	66.6		
*BN with history of AN*											
Spine BMD	4	67	21	− 0.74	− 1.06	− 0.43	4.58	** < 0.001**	0		
*BN without history of AN*											
Spine BMD	2	85	96	− 0.07	− 1.05	− 0.90	0.14	0.885	89.7		
*OSFED/EDNOS only*											
Total body BMD	1	22	34	− 0.04	− 0.99	0.10	1.60	0.109			
Spine BMD	1	22	34	− 2.44	− 3.15	− 1.73	6.77	** < 0.001**			
Femur BMD	1	22	34	− 0.24	− 0.76	0.31	0.82	0.415			

P-value in bold indicates statistical significance (*p* < 0.05)

*N* sample number, *ED* eating disorder, *HC* healthy control, *SMD* standardised mean difference, *L* lower, *U* upper, *CI* confidence interval, *Z* z-scores, *p* *p* value, *BMD* bone mineral density, *AN* anorexia nervosa, *BN* bulimia nervosa, *OSFED* other specified feeding or eating disorder, *EDNOS* Eating disorder not otherwise specified, *I*^*2*^ Higgins I^2^ test

#### BMD in AN

The AN group had lower BMD than the HC in all the anatomical sites i.e. total body BMD (SMD = − 3.45 [− 4.57 to − 2.33], *p* < 0.001), spine BMD (SMD = − 4.34 [− 5.27 to − 3.40], *p* < 0.001), femur BMD (SMD = − 3.38 [− 5.29 to − 2.21], *p* < 0.001) and hip BMD (SMD = − 4.95 [− 6.78 to − 3.12], *p* < 0.001).

#### BMD in BN

The BN group had lower total body (SMD = − 0.29 [− 0.46 to − 0.12], *p* = 0.001)] and spine BMD (SMD = − 0.49 [− 0.75 to − 0.21], *p* = 0.001) than the HC group, respectively. However, the three studies included, found no differences at the femur (SMD = − 0.19 [− 0.80 to − 0.43], *p* = 0.552). Among those with BN (with or without previous history of AN), when compared to HC, only the group with a previous diagnosis of AN had significantly lower spine BMD (SMD = − 0.74 [− 1.06 to − 0.43], *p* < 0.001)].

#### BMD in OSFED/EDNOS

In the only study included, the OSFED/EDNOS group had lower spine BMD than HC (SMD = − 2.44 [− 3.15 to − 1.73], *p* < 0.001)]. No differences were found at total body BMD (SMD = − 0.04 [− 0.99 to 0.09], *p* = 0.109) and femur BMD (SMD = − 0.24 [− 0.76 to 0.31], *p* = 0.415).

### Meta-regression results

Analysis of the whole EDs group (Table 3), showed that fat mass and lean mass were significantly predictive of a difference in total body BMD (β = 0.24; 95% CI 0.09–0.39; *p* = 0.002 and β = 0.41; 95% CI 0.037–0.78; *p* = 0.03, respectively). Both BMI (β = 0.74; 95% CI 0.11–1.38; *p* = 0.02) and fat mass (β = 0.26; 95% CI 0.04–0.48; *p* = 0.02) were positively associated with higher spine BMD but not lean mass. Presence of amenorrhoea was negatively associated with total body BMD (β = − 0.04; 95% CI − 0.07 to 0.01; *p* = 0.01) and spine BMD (β = − 0.04; 95% CI − 0.07 to − 0.01; *p* = 0.01).

**Table 3:** Meta-regression results of covariables effect on total, spine, femur and hip bone mineral density

Covariate	Total body BMD				Spine				Femur				Hip			
	β	L 95% CI	U 95% CI	*p*	β	L 95% CI	U 95% CI	*p*	β	L 95% CI	U 95% CI	*p*	β	L 95% CI	U 95% CI	*p*
*All studies*																
Age	0.08	− 0.15	0.32	0.485	0.27	0.00	0.55	0.050	0.19	− 0.30	0.69	0.443				
Illness duration (yr)	0.16	− 0.26	0.58	0.460	0.30	− 0.14	0.75	0.178	− 0.03	− 0.53	1.06	0.513				
BMI (kg/m^2^)	0.50	− 0.11	1.01	0.055	0.74	0.11	1.38	**0.022**	0.66	− 0.40	1.72	0.220				
Fat mass (kg)	0.24	0.09	0.39	**0.002**	0.26	0.04	0.48	**0.022**	0.07	− 0.21	0.35	0.632				
Lean mass (kg)	0.41	0.04	0.78	**0.031**	0.45	− 0.10	1.01	0.110	0.30	− 0.30	0.91	0.322				
Amenorrhea (%)	− 0.04	− 0.08	− 0.01	**0.010**	− 0.04	− 0.07	− 0.01	**0.011**	− 0.03	− 0.08	0.02	0.214				
Amenorrhea duration (mo)	0.03	− 0.03	0.09	0.380	0.04	− 0.04	0.11	0.347	− 0.99	− 2.15	0.17	0.095				
OCP use (%)	0.01	− 0.05	0.07	0.711	0.03	− 0.03	0.08	0.376	0.04	− 0.04	0.13	0.324				
*AN only*																
Age	− 0.06	− 0.36	0.24	0.698	0.19	− 0.17	0.55	0.309	0.16	− 0.46	0.78	0.610	0.10	− 0.37	0.56	0.684
Illness duration (yr)													0.15	− 1.03	1.34	0.804
BMI (Kg/m^2^)	0.17	− 1.13	1.47	0.797	0.61	− 1.19	2.40	0.506	1.46	− 0.75	3.67	0.196	0.55	− 1.92	3.02	0.661
Fat mass (kg)	0.32	0.05	0.60	**0.021**	0.22	− 0.19	0.64	0.291	− 0.07	− 0.50	0.37	0.768	− 0.24	− 0.85	0.38	0.445
Lean mass (kg)	0.38	− 0.13	0.89	0.141	0.37	− 0.38	1.12	0.336	0.21	− 0.60	1.03	0.610	0.48	− 0.59	1.55	0.379
Amenorrhea (%)													0.01	− 0.09	0.11	0.860
Amenorrhea duration (mo)	− 0.17	− 0.46	0.12	0.250									− 0.07	− 0.50	0.36	0.747
OCP use (%)													− 0.04	− 0.16	0.10	0.564

P-value in bold indicates statistical significance (*p* < 0.05)

*BMD* bone mineral density, *β* coefficient, *L* lower, *U* upper, *CI* confidence interval, *p* *p*-value, *AN* anorexia nervosa, *yr* years, *mo* months, *OCP* oral contraceptive pill

In individuals with AN, fat mass was significantly associated with total body BMD (β = 0.32; 95% CI 0.04–0.60; *p* = 0.02), but not with spine, femur, and hip BMD. Meta-regression analyses were not conducted on the BN and OSFED/EDNOS groups due to the limited number of studies measuring the predictors of interest.

### Sensitivity analyses

**Fig. 4:**
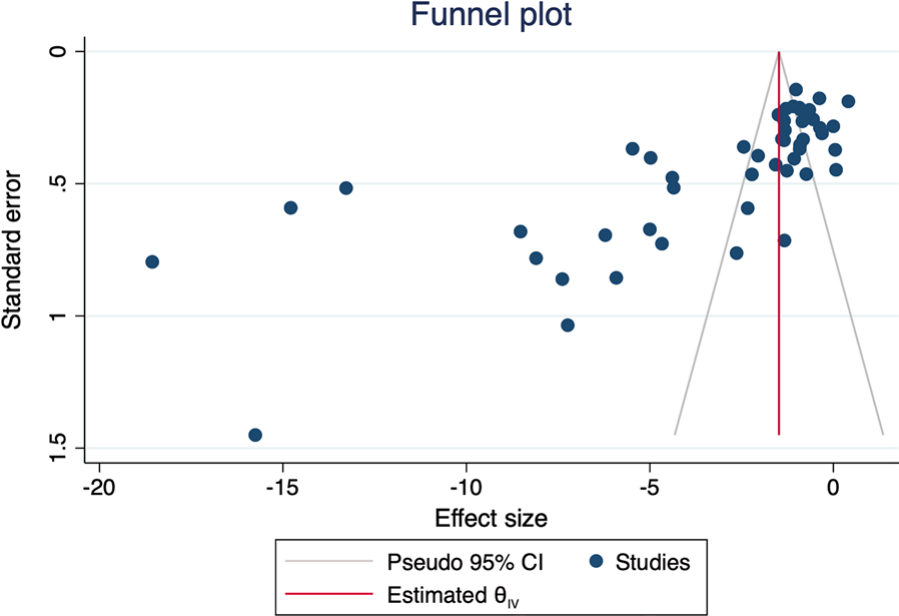
Funnel plot of spine bone mineral density studies included in the meta-analysis

The Higgins I^2^ test of total body BMD meta-analyses indicates high heterogeneity of the total ED group (99.2%) and the AN group (99.1%) but not of the BN group (4.8%). Heterogeneity of spine analyses was high for all EDs (I^2^ = 99.4%), AN (I^2^ = 99.4%) and BN (I^2^ = 63.7%) groups. Funnel plots (Fig. 4) and Egger’s test (t = − 6.87, *p* =  < 0.001) suggest publication bias in meta-analyses on spine BMD.

## Discussion

### Summary of findings

We examined spine, hip, femur, and total body BMD data and identified key body composition and menstrual status predictors of the difference between EDs and HC. Due to the limited number of studies, absence of papers and/or measurements of predictors of interest, our meta-analyses and meta-regressions of total, spine and femur BMD included individuals with AN, BN and OSFED/EDNOS and that of the hip BMD included individuals with AN only. The quality of the studies included was good with the majority of studies controlling or adjusting comparisons with HC for age.

Whilst in people with AN lower BMD than HC at all sites is consistent with previous reviews [6, 14, 15], what we have found is that lower total body, spine and femur BMD is not limited to AN. Specifically, a history of AN and having amenorrhoea may increase the risk of low BMD in individuals with BN and OSFED.

Our study helps clarify the somewhat inconsistent literature on BMD in women with BN in cross-sectional studies [53, 64] and previous meta-analyses [6, 14]. In individuals with BN, BMD was lower than in HC, at total body and in the spine. However, in a subgroup analysis of studies reporting the presence or absence of a history of AN, only those with a history of AN had lower spine BMD than HC. This is in accord with previous reports [14]. Only five of the studies with BN assessed whether there was a history of AN and 36% of the individuals included in the OSFED/EDNOS group reported a history of AN. This raises the question whether individuals with BN/OSFED without a history of AN are at risk for impaired bone health [14]. There is evidence from the ALSPAC study suggesting that not only a lifetime history of AN but also having ED behaviours (i.e. fasting and food restriction) are associated with a reduction in BMD by middle-adulthood [69]. Therefore, measurement of BMD should not be limited to those with a current AN diagnosis, but also be made in individuals with a history of AN, irrespective of their current ED diagnosis.

When AN, BN and OSFED/EDNOS groups were assessed as one ‘ED’ group, the greatest magnitude of the difference in BMD was at the spine. This is likely to be driven by the AN and OSFED/EDNOS groups (both with a mean BMI < 18.5 kg/m^2^), i.e. there were no significant differences at the femur when BN and HC groups were assessed alone. In the AN group compared to HC, SMD at the hip and spine were greater than at the femur and total body. In the BN group versus HC, BMD was lower at the spine than in the total body. However, the majority of studies reported spine BMD, with fewer measuring total body BMD and, thus these two findings cannot be objectively compared. Spine BMD was lower than in HC in OSFED/EDNOS participants in the single study included with this diagnosis. While BMD at the hip was lowest in the AN group compared to HC, ~ 30% of studies involving participants with AN reported this measure. Oestrogen deficiency is more likely to affect trabecular bone, which can be detected by loss of spine BMD [70], while aging and peak mass accrual would more significantly affect cortical bone and be detected by hip and femur BMDs [71, 72]. In people with EDs, there is a need for a more consistent assessment of bone anatomical sites to understand the pathophysiology and the specific risk of fractures and pain at different parts of the skeleton.

### Associations between body composition, menstrual health and BMD in EDs

Low BMI was a predictor of lower BMD in the whole group. Calorie restriction results in extensive weight loss in AN [73] and associations between low BMI and lower BMD, reported previously [14, 15, 74], are supported by our study.

Calorie restriction in people with EDs could be a potential predictor of BMD loss. Energy deficits lead to hormonal changes, including decreased insulin-like growth factor-1 (IGF-1), growth hormone (GH) resistance, amenorrhoea, increased cortisol levels, and changes in hunger and satiety signalling (due to decreased levels of leptin, increased peptide YY, and ghrelin resistance) [75–78]. In vitro and in vivo studies in EDs suggest an ‘uncoupling’ of bone turnover, with increased osteoclastic bone reabsorption activity in comparison to bone formation activity by osteoblasts [79]. In addition, calorie restriction or starvation in animal studies and in individuals with AN has been shown to increase marrow adipose tissue (MAT) in the bone [80, 81]. As MAT cells derive from the same lineage as osteoblast precursors cells [82, 83], it is speculated that MAT expansion in individuals with AN could potentially act as an ‘emergency storage’ of adipocytes to facilitate survival during starvation but leading to bone weakness [84].

Although caloric restriction is an essential feature for a diagnosis of AN, the study of Elran-Barak et al. [85] showed that individuals with AN and BN did not differ in terms of fasting, number of meals per day, very small meals and low-calorie meals. The authors suggest that a subset of people with BN (who are able to maintain dietary restriction, low frequency of binge eating and ingesting high calorie foods only during binge episodes) are prone to inadequate nutrition. In our study, total and spine BMD were lower in the BN group than in HC, i.e. behaviour resulting in calorie restriction in people with BN may be associated with loss of BMD.

Low fat mass was a predictor of lower BMD in the whole group and in the AN group. This could be related to decreased plasma leptin levels [86, 87], especially because women with AN tend to lose more peripheral (subcutaneous, extremity) than central (visceral, trunk, android) fat [88, 89]. However, low weight does not necessarily indicate low fat mass in all AN patients as it is also due to decreased muscle, organs and bone mass [90]. Lean mass was a predictor for lower BMD in individuals with EDs. However, we did not confirm previous evidence of a lower lean mass in AN being associated with decreased BMD [15]. Physical exercise (especially load-bearing) is important for gaining lean mass, and for achieving and maintaining peak bone mass in adults. However, the protective or detrimental effects of physical activity on BMD remain controversial in the ED field, where individuals are at a higher risk of excessive exercise [91]. People at very low weight, and with amenorrhoea, may be advised to limit physical activity and avoid high-impact activities that increase the chance of falls and injuries [18].

Our findings provide support for the proposal that body composition and history of lowest-ever and highest-ever BMI, in addition to BMI, should be used to determine individuals’ risk for low BMD and osteoporosis [92]. This could be particularly important for individuals with a history of AN, whose current body composition does not necessarily suggest that they have lowered BMD. Non-invasive, easy-to-operate, and reasonably accurate methods such as BIA could be used repeatedly to track individuals' changes in fat and lean mass before, during, and after ED treatment.

Despite previous evidence, we found that duration of amenorrhoea, age and illness duration were not significant predictors of BMD in our meta-analysis. This may reflect the fact that menstrual function and ED history were not well reported in many studies, and the lack of a standardised definition for estimating illness duration. Of the 61 groups with EDs included in the review, duration of amenorrhoea was reported in only 19 (31%) of them. As these were predominantly younger participants, the duration of both illness (M = 5.8 years, SD = 3.7) and amenorrhea (M = 23.9 months, SD = 28.7) were shorter, and for this reason they did not significantly associate with BMD. However, the percentage of participants with amenorrhoea was negatively related to total body and spine BMD, which was reported by part of the sample in 5 of the 12 studies with BN participants.

Any interruption of menstruation for a prolonged period results in bone loss and is the main determinant of osteoporosis risk in women [93]. The teenage years have the highest incidence of EDs [1] and are a critical growth period, when oestrogen has a role in the closure of epiphyseal growth plates [94]. Although resumption of menses in adult women with AN was a predictor of BMD recovery [76], the evidence of reduced BMD in both females with eumenorrhoea and amenorrhoea with a similar BMI [95] shows that low levels of oestrogen cannot solely explain the severity of bone loss in AN [76]. Therefore, longitudinal investigation of the relationship between ED onset and severity, menstrual function, and bone mass accrual is necessary to identify to what extent the ED impairs BMD and whether this can be reversed after ED recovery and throughout life.

In addition, there are not sufficient data to draw conclusions of how the exclusion of amenorrhoea from diagnostic criteria for AN in DSM-5 has affected differences in BMD of individuals with AN in comparison to HC. DSM-IV was the main method for AN diagnosis in the studies included and only 4 of them used DSM-5 [29, 33, 59, 68]. In these studies, participants with AN had mean a BMI varying from 14.4 to 17.5 kg/m^2^ [29, 33, 59, 68] but in the study by Wu et al. [68] all participants were amenorrheic. OCP use was reported by 15.2% of participants in the study by Schorr et al. [59] only. Although there is consensus that the use of OCP is not indicated for the purpose of preventing bone loss in EDs [96], the lack of studies reporting it did not allow us to systematically assess it as a predictor of BMD in this ED group.

**Fig. 5:**
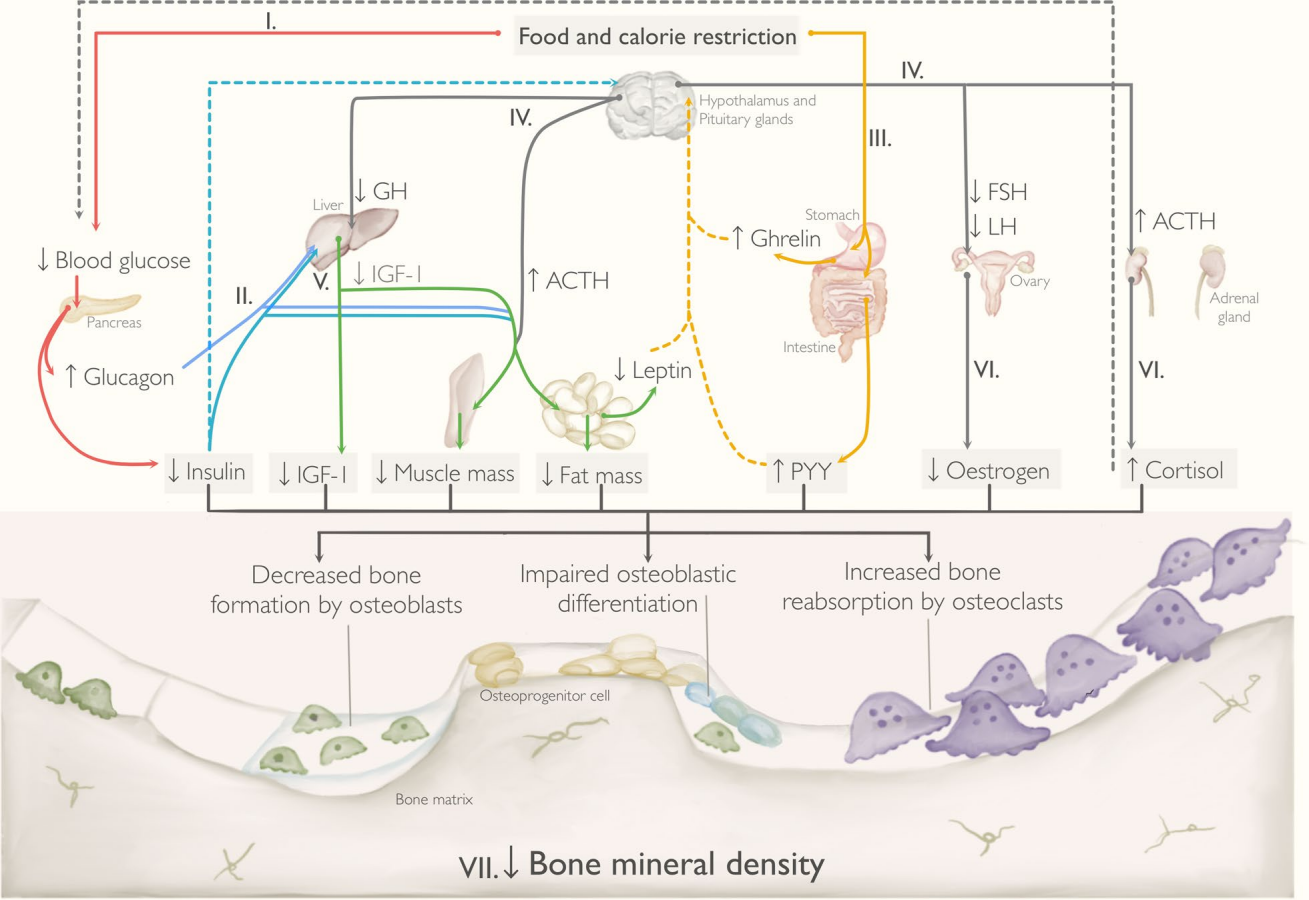
Model showing interactions between body composition, hormonal changes and decreased BMD in individuals with EDs, where dietary restriction leads to a negative energy balance. Legend: This figure illustrates potential mechanisms for interactions between body composition, hormonal changes and BMD loss in females with EDs, where dietary restriction leads to a negative energy balance. (I) Food and calorie restriction leads to lower levels of glucose in the blood, reducing insulin and increasing glucagon secretion by the pancreas. (II) Changes in insulin and glucagon hormones enhance lipolysis, and reduce glycogen synthesis and glucose uptake by the muscle. (III) In EDs, altered hunger and satiety signalling is marked by reduced levels of leptin (due to a diminished adipose tissue), increased peptide YY (PYY), and ghrelin resistance. (IV) All of this, in addition to the lower levels of insulin, inhibit the hypothalamic–pituitary–gonadal axis and lead to reduced levels of growth hormone (GH), follicle-stimulating hormone (FSH), luteinizing hormone (LH) and increased levels of adrenocorticotropic hormone (ACTH). (V) As a consequence, low GH has catabolic effects on muscle mass, fat mass and bone mineral density, via insulin-like growth factor-1 (IGF-1). (VI) Lower FSH and LH levels reduce oestrogen production by the ovaries and ACTH stimulates cortisol production by the adrenal glands. (VII) Decreased insulin, IGF-1, muscle mass, fat mass, oestrogen, and increased PYY and cortisol negatively affect the rate of bone formation and reabsorption by osteoblasts and osteoclasts, respectively

Figure 5 proposes an explanatory model of the possible interactions between body composition, hormonal changes and bone remodelling in people with EDs in which dietary restriction leads to a negative energy balance.

Osteopenia and osteoporosis in people with EDs are difficult to treat. Bone loss may not be completely reversible even after ED recovery, and nutritional supplements and oral contraceptives do not significantly increase BMD [17]. For people with long-term low body weight and low BMD, NICE guidelines consider the use of transdermal 17-β-oestradiol with cyclic progesterone (by young women between 13 and 17 years with a bone age lower than 15 years) and of bisphosphonates (by adult women 18 + years after discussing benefits and risks, i.e. teratogenic effects) [18]. Weight gain and menses resumption remain the first line of treatment for decreased BMD in individuals with EDs [17].

Repeated DXA scans (no more than once per year) are recommended for individuals with ongoing and persistent underweight (after 1 year in children and adolescents, 2 years in adults or earlier if bone pain and/or recurrent fractures are observed) [18]. The International Osteoporosis Foundation recommends that the skeletal assessment should include a comprehensive history and complete physical examination [97]. Therefore, understanding the interplay between body weight, fat mass, lean mass, menstrual function, and BMD changes across the various ED diagnoses can help identify those most at risk for osteoporosis and provide targeted and early intervention. Bone studies in EDs should (when possible) assess menstrual function and history, providing relevant information including number of participants with amenorrhoea, menarchal age, date of last menstrual period, current and history of OCP/other hormonal therapy use.

## Strengths and limitations

We systematically reviewed the current literature assessing BMD in individuals with EDs (AN, BN, BED and EDNOS/OSFED) versus HC. To the best of our knowledge, this is the first review where age, illness duration, amenorrhoea, OCP use, BMI, fat mass, lean mass, and history of AN were investigated in the same meta-analysis as predictors for BMD difference for all EDs group. Studies with one or more eligible samples for the same ED diagnosis had the characteristics of each group preserved (ED subtype, menstrual status, OCP use, history of AN, diagnosis method) and outcome measures were included independently. Our findings provide strong evidence for conducting bone health assessments in individuals with a history of AN, irrespective of current ED diagnosis.

However, there was a limited number of papers with outcomes of interest for OSFED/EDNOS and BED derived from this review, and the only study focused on EDNOS did not allow a comparative analysis of BMD in those with or without a history of AN. Therefore, independent of the anatomical site, differences in BMD between EDs group and HC in OSFED/EDNOS were mostly driven by the AN group. Studies with BED did not meet the criteria for inclusion in this systematic review. The majority of studies did not use a structural clinical interview for distinguishing controls from individuals with EDs and bias in the selection of control may have influenced the accuracy of results. The results of our analyses were not controlled for race/ethnicity, symptom severity and variation in DXA machines. Therefore, the results of this present meta-analysis cannot be generalised beyond the populations studied within this review.

Our review includes studies with publication dates from 1989 to 2022. During this period several editions of DSM were published, and ED criteria changed between versions. However, except for amenorrhoea (which was removed in DSM-5 from the AN criteria) all the main elements of the diagnosis of an ED that might potentially affect BMD, remained relatively consistent across the editions (low weight/weight loss, food restriction, fear of weight gain, compensatory and purging behaviours).

Participants could not be classified and analysed separately according to menstrual status or illness duration in this meta-analysis. Lastly, physical activity data were not included in our review: as this is linked to protective and risk factors in relation to BMD, it could have helped clarify the effects of lean mass on BMD.

## Conclusions

This review found lower BMD (total body, spine, and femur) in individuals with EDs (AN, BN and OSFED/EDNOS). In those with a diagnosis of BN, the lower BMD (especially in the spine), may be due to a history of AN. Secondly, individuals with AN are at more risk of diminished bone health in their spine and hip rather than in other regions. Ideally, assessing BMD in the four main anatomical sites will provide a more global picture of the effect of an ED on vulnerabilities in different bone regions and to what extent changes in BMD in different regions are/can be reversed following recovery.

Meta-regression analyses showed that low BMI, low fat mass, low lean mass and being amenorrhoeic are predictors of lower BMD in people with an ED. In those with AN, low fat mass was the only predictor of low spine BMD. Therefore, individuals with current or past AN should have their bone health assessed. To make more accurate assessments of individual risk of low BMD and osteoporosis, investigations should include measures that help predict body composition, menstrual function and history, physical exercise, and energy metabolism hormones.

## Data Availability

The datasets used and/or analysed during the current study are available from the corresponding author on reasonable request.

## Abbreviations

AN: Anorexia nervosa
AN-BP: AN-Binge Purge subtype
AN-BN: AN with an additional diagnosis of BN (according to DSM-III-R)
AN-R: AN-Restrictive subtype
BED: Binge eating disorder
BIA: Bioelectrical impedance analysis
BMD: Bone mineral density
BMI: Body mass index
BN: Bulimia nervosa
BN-NPAN: BN with no previous AN history
BN-PAN: BN with previous AN
CT: Computerised tomography
DPA: Dual photon absorptiometry
DSM: Diagnostic and Statistical Manual of Mental Disorders
DXA: Dual-energy X-ray absorptiometry
EDNOS: Eating disorder not otherwise specified
EDs: Eating disorders
GH: Growth hormone
HC: Healthy controls
HPG: Hypothalamic–pituitary–gonadal axis
ICD: International Classification of Diseases
IGF-1: Insulin-like growth factor-1
MAT: Marrow adipose tissue
MRI: Magnetic resonance imaging
NOS: Newcastle–Ottawa scale
OCP: Oral contraceptive pill
OSFED: Other specified feeding or eating disorder
PYY: Peptide YY
SMD: Standardised mean difference
